# Editorial: Phosphonate Chemistry in Drug Design and Development

**DOI:** 10.3389/fchem.2021.695128

**Published:** 2021-04-30

**Authors:** Petri A. Turhanen, Konstantinos D. Demadis, Pawel Kafarski

**Affiliations:** ^1^ School of Pharmacy, University of Eastern Finland, Kuopio, Finland; ^2^ Crystal Engineering, Growth and Design Laboratory, Department of Chemistry, University of Crete, Heraklion, Greece; ^3^ Department of Bioorganic Chemistry, Wroclaw University of Science and Technology, Wroclaw, Poland

**Keywords:** organophosphorus, phosphonate, drug targeting, nucleotides, prodrug, stereoselective, bisphosphonate

**FIGURE 1:**
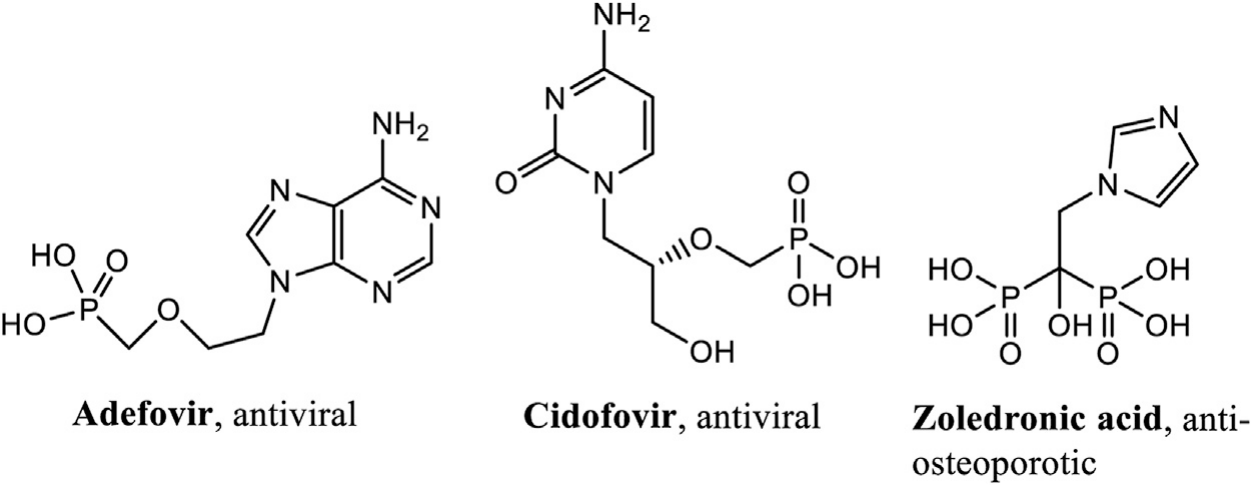
Examples of phosphonate drugs of medicinal use.

Phosphonates are surrogates of natural phosphates, but they possess direct C-P bonds and one or more phosphonic acid moieties (R-PO_3_H_2_). These compounds are also generally used as a bioisosteres of carboxylates and serve as analogs of carboxylic acids, amino acids and peptides. Phosphon(in)ate groups are also actively mimicking the transition state during hydrolysis of amides and esters, thus being effective transition-state inhibitors. The interest in these compounds has grown immensely because of: their bioactive properties (as drugs, pro-drugs), their use as the tools for the design of enzyme inhibitors, their potential as bone-targeting drugs, their novel antibacterial and anticancer activity, their use in medical imaging and diagnostics and their phosphoantigen properties. There are several compounds containing phosphonate groups that are already in use as antiviral drugs. For example, bisphosphonates, which are active drugs against osteoporosis, demonstrate rich chemistry and biology. Applications of phosphonates cover a plethora of research fields, beyond the realm of medicinal chemistry. Examples of phosphonate compounds currently in medicinal use are given in Figure 1.

In the current research topic three review articles were published covering: antiviral and anticancer pro-nucleotides, phosphonate and bisphosphonate inhibitors of farnesyl pyrophosphate synthases, and biologically active nucleoside phosphonates.

Kraszewski et al. presented a short account of their work on the synthesis and biological activity of electrically neutral and charged anti-HIV and anticancer pronucleotides, presented in the framework of contemporary research in this area.

Phosphonates and bisphosphonates have proven their pharmacological utility as inhibitors of enzymes that metabolize phosphate and pyrophosphate substrates. Nitrogen-containing bisphosphonates represent the best-known examples. Widely used to treat bone-resorption disorders, these drugs mostly work by inhibiting the enzyme farnesyl pyrophosphate synthase. Playing a key role in the isoprenoid biosynthetic pathway, this enzyme is also a potential anticancer target. A comprehensive overview was presented on research efforts to identify new inhibitors of farnesyl pyrophosphate synthase for various therapeutic applications. While the majority of these efforts have been directed against the human enzyme, some have been targeted on its homologs from other organisms, such as protozoan parasites and insects. The main focus is on the structures of the target enzymes and how the structural information has guided the drug discovery efforts (Park et al.).

The use of the phosphonate motif featuring a C-P bond as bioisosteric replacement of the labile P–O bond is widely recognized as an attractive structural concept in different areas of medicinal chemistry, since it addresses the very fundamental principles of enzymatic stability and minimized metabolic activation. This review aims to guide readers through the fundamentals of nucleoside phosphonate therapeutics in order to inspire the future design of molecules to target infections that are refractory to currently available therapeutic options (Groaz and Jonghe).

Four original research papers published are related to: (*S*)-thienyl and (*R*)-pirydyl phosphonate derivatives synthesized by stereoselective resolution, synthesis of the phosphonopropionic acid derivatives as potential inhibitors of Rab geranylgeranyl transferase, selective inhibition of 2-oxoglutarate and 2-oxoadipate dehydrogenases by the phosphonate analogs of their 2-oxo acid substrates, and highly stereoselective synthesis of α-fluoro- β- or γ-amino alcohol derivatives of alkylphosphonates.

*Rhodotorula mucilaginosa* was successfully applied as a biocatalyst for the enantioselective resolution of the racemic mixtures of heteroaromatic aminophosphonates. The biological synthesis provided effectively both enantiomers of these products. The scale was enlarged to semi-preparative stage, using a simplified flow-reactor and immobilized biocatalyst (Katarzyna Lubiak-Kozłowska).

Twelve phosphonopropionates derived from 2-hydroxy-3-imidazo[1,2-a]pyridin-3-yl-2-phosphonopropionic acid (3-IPEHPC) were synthesized and evaluated for their activity as inhibitors of protein geranylgeranylation. The nature of the substituent in the C6 position of imidazo[1,2-a]pyridine ring was responsible for the compound’s activity against Rab geranylgeranyl transferase (RGGT). The most active inhibitors disrupted Rab11A prenylation in the human cervical carcinoma HeLa cell line. The esterification of carboxylic acid in the phosphonopropionate moiety turned the inhibitor into an inactive analog (Kusy et al.).

Phosphonate analogs of pyruvate and 2-oxoglutarate are established as specific inhibitors of cognate 2-oxo acid dehydrogenases. The present work develops this class of compounds as specific *in vivo* inhibition of 2-oxoglutarate dehydrogenase (OGDH) and its isoenzyme, 2-oxoadipate dehydrogenase (OADH). Phosphonate analogs of C5-C7 dicarboxylic 2-oxo acids inhibit OGDH and OADH competitively to 2-oxo substrates in all sites. The consistent kinetic and structural results expose adipoyl phosphonate as a valuable pharmacological tool for specific *in vivo* inhibition of the DHTKD1-encoded OADH, a new member of mammalian family of 2-oxo acid dehydrogenases, up-regulated in some cancers and associated with diabetes and obesity (Artiukhov et al.).

Rapp et al. reported the synthesis of stable surrogates of an important amino acid (*R*)-4-amino-3-hydroxybutyric acid (GABOB) such as substituted hydroxy aminophosphonic acids bearing quaternary stereogenic center. Highly diastereoselective syntheses of fluorinated spiroepoxyalkylphosphonate or related tertiary carbon-containing oxiranes from β-ketophosphonates possessing methyl, phenyl, or cyclohexenyl substituents, were reported. Stereoselective acid-promoted epoxide opening by bromide or azide followed by the reduction/protection afforded tertiary bromides or N-Boc derivatives of β-amino-γ-hydroxyalkylphosphonates in most cases, while the reactions of oxiranes with different amines yielded their β-hydroxy-γ-amino regio-isomers. Surprisingly, during the synthesis of amino phosphonic acids, the acid-induced rearrangement proceeded in a high diastereospecific manner, leading finally to substituted β-hydroxy-γ-aminoalkylphosphonic acids (Rapp et al.).

The “world” of organophosphorus chemistry is constantly expanding. Whether in “simple” or more “complex” molecules, the presence of the phosphonate group endows these compounds with exciting features that are being exploited in the fields of medicinal chemistry, organic chemistry, pharmacology and several other interdisciplinary areas. We strongly encourage researchers to get familiar with the fascinating “world” of organophosphorus chemistry and discover its limitless potential.

